# A study protocol for a pilot randomized controlled trial to evaluate the effectiveness of a gene-based nutrition and lifestyle recommendation for weight management among adults: the MyGeneMyDiet^®^ study

**DOI:** 10.3389/fnut.2023.1238234

**Published:** 2023-08-22

**Authors:** Jacus S. Nacis, Jason Paolo H. Labrador, Diana Glades D. Ronquillo, Marietta P. Rodriguez, Aurora Maria Francesca D. Dablo, Ruby D. Frane, Marilou L. Madrid, Noelle Lyn C. Santos, Julianne Janine V. Carrillo, Mikko Glen Fernandez, Gerard Bryan L. Gonzales

**Affiliations:** ^1^Department of Science and Technology – Food and Nutrition Research Institute (DOST-FNRI), Taguig, Philippines; ^2^Division of Human Nutrition and Health, Wageningen University & Research, Wageningen, Netherlands; ^3^Department of Public Health and Primary Care, Faculty of Medicine and Health Science, Ghent University, Ghent, Belgium

**Keywords:** genotype, lifestyle genomics, nutrigenomics, nutritional genomics, obesity, overweight

## Abstract

**Introduction:**

Managing nutrition and lifestyle practices, nutrition phenotypes, and the genome forms the foundation of precision nutrition. Precision nutrition focuses on metabolic variability among individuals, and one approach to achieving its goals is to integrate gene-based nutrition and lifestyle recommendations in nutrition practice. However, scientific evidence proving the effectiveness of such recommendations is limited. This study will examine whether providing nutrition and lifestyle recommendations based on individual genotype can lead to better weight loss, along with reduction in body mass index (BMI), waist circumference, and body fat percentage among overweight and obese adults.

**Methods and analysis:**

A parallel group, single-blind, randomized controlled trial will be conducted. Sixty-two overweight/obese individuals aged 19–59 years old will be recruited. Participants will be randomly allocated to either the intervention (*n* = 31) or the control arm (*n* = 31). Participants in the intervention group will receive the MyGeneMyDiet^®^ Recommendation for Weight Management, a gene-based nutrition and lifestyle recommendation that was developed based on existing evidence of the effects of *FTO* rs9939609 on body weight, BMI, and physical activity; *UCP1* rs1800592 on calorie intake; and *TCF7L2* rs7903146 on dietary fat intake. Participants in the control group will receive the standard recommendations for weight management. The primary outcomes will be the differences in weight, BMI, waist circumference, and body fat percentage between arms in both the active phase (6 months) and inactive phase (last 6 months) of the trial. Participants in both arms will be evaluated at baseline and in months 3, 6, 9, and 12.

**Discussion:**

To the best of our knowledge, this will be the first gene-based intervention that will adopt a phase of intensive nutrition counseling, followed by a simulation of a free-living state to determine adherence to a gene-based recommendation. This study will contribute to the future implementation of precision nutrition interventions by providing evidence on the effectiveness of a gene-based nutrition and lifestyle recommendation for weight loss.

**Clinical trial registration:**

clinicaltrials.gov, identifier [NCT05098899].

## 1. Introduction

Genetics is one of the factors that influence the variability of responses to weight-loss interventions (1). Certain genetic polymorphisms are believed to affect an individual’s tendency toward obesity and other-related aspects such as appetite control and energy balance (2–4). For instance, the risk allele of *FTO* rs9939609 closely relates to obesity and other related phenotypes in various populations (5–9). Earlier studies have observed the influence of physical activity in this genetic variant, as demonstrated by the significantly higher obesity risk among physically inactive carriers of the risk allele (8–10), and the reduction of such risk among the physically active risk allele carriers (9, 11). On the other hand, mutations in the *UCP1* gene contribute to the development of obesity by reducing energy expenditure modulating the thermogenic function on brown adipose tissue (12, 13). The variant rs1800592 (also known as the -382A/G mutation) is associated with resistance to weight loss in response to energy restriction (14). Carriers of the risk allele for this variant demonstrated lesser weight loss from controlled-energy diet regimen (14, 15), even when exercise is an added regimen to the energy-restricted diet (16). Obese women carrying the ht3 [GAG] haplotype showed accelerated reduction of waist-to-hip ratio and body fat mass when a very low-calorie diet (700 kcal/day) was given to them (17). Moreover, it is becoming apparent that obesity mediates the strong association of *TCF7L2* rs7903146 with type 2 diabetes (18). This variant is associated with obesity risk (19), obesity phenotype (20–22), and several obesity comorbidities such as impaired glucose homeostasis and increased lipid and C-reactive protein levels (23). Dietary intake of saturated fat appears to augment the risk of metabolic syndrome (20) as individuals who carry the risk allele of rs7903146 have better responses to weight loss when fed with a low-fat diet (18). High dietary saturated fat intake accentuates the effect of the variant, implying that dietary fatty acid consumption potentially modifies genetic susceptibility toward metabolic syndrome (20).

Earlier studies have shown that dietary advice based on genetic information resulted in specific changes in the intake of sodium and dietary fat (24, 25). However, substantial research showing the effectiveness of gene-based nutrition recommendations for improving weight and obesity-related outcomes are rather limited. Available evidence showed none or modest improvements in weight, lifestyle, and dietary behavior when advice was linked to genetic profiles (24, 26–28) or when genetic risks were disclosed to the participants (24, 25, 29, 30).

To expand the existing knowledge about the effects of gene-based nutrition and lifestyle recommendations, this randomized controlled trial aims to determine if the provision of the MyGeneMyDiet^®^ Recommendation for Weight Management and disclosure of genetic risk can help overweight/obese individuals achieve 5–10% weight loss in a 12-month trial when compared with the standard advice for weight management. Other primary outcomes include reduction in BMI, waist circumference, and body fat percentage.

## 2. Methods and analysis

### 2.1. Study design

**Figure 1:**
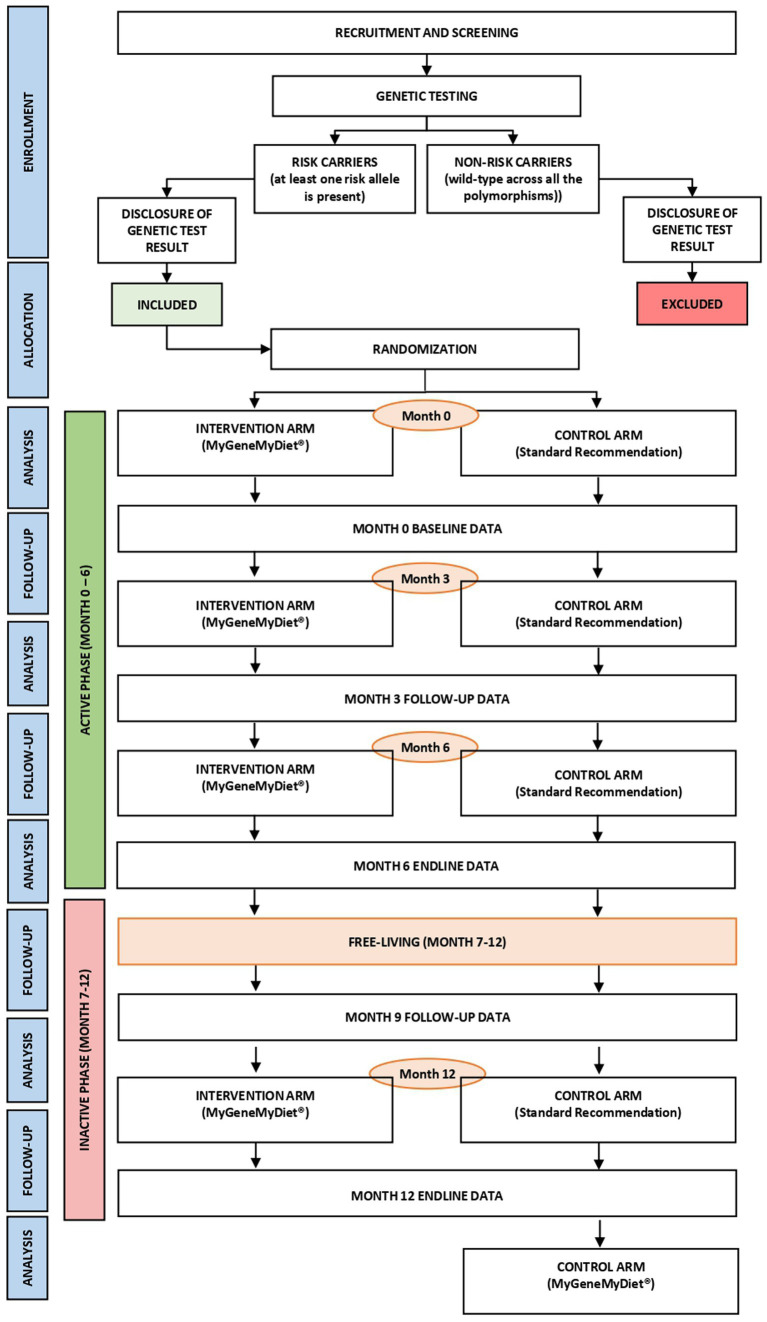
The design of the MyGeneMyDiet^®^ Study.

The MyGeneMyDiet^®^ study is a parallel-group, single-blind, randomized controlled trial (Figure 1). Sixty-two overweight or obese adults will be recruited through waves of study enrollment. The 12-month trial will entail an “active” phase (first 6 months), followed by an “inactive” phase from months 7–12. During months 1–6, all participants will receive three sessions of nutrition counseling, including general health and information presented in nutrition modules designed for the study. A simulation of a “free-living state” will be applied to the participants during months 7–12. During this period, a follow-up data collection on month 9 and the nutrition counseling session at the end of the study on month 12 will be initiated. The inactive phase will determine whether the participants continue to adhere to the dietary and lifestyle recommendations given to them during the active phase.

### 2.2. Recruitment and informed consent processes

Invitation letters, social media promotions, and snowball sampling will be employed to recruit participants. Social media promotional materials will contain electronic links and contact information of the research team. Orientation sessions will be conducted via videoconferencing platforms such as Zoom webinars. Details of the study protocol and the informed consent form, including an open forum to tackle the rights and privileges of the participants, will be discussed during these sessions. The informed consent form will be emailed to the prospective study participants after the orientation.

### 2.3. Eligibility criteria

#### 2.3.1. Inclusion criteria

The trial targets overweight or obese individuals aged between 19 and 59, who indicate willingness to participate in the study. Their BMI should be equal to or greater than 25 but not more than 40 kg/m^2^. They should also have normal to pre-disease state level of fasting blood glucose, lipid profile, and blood pressure, and blood cortisol and thyroid hormone levels. They should carry at least one of the risk alleles of the three target genetic polymorphisms: A allele for *FTO* rs9939609, T allele for *TCF7L2* rs7903146, and G allele for *UCP1* rs1800592.

#### 2.3.2. Exclusion criteria

The exclusion criteria are as follows: individuals with elevated levels of fasting blood glucose, lipids, blood pressure, thyroid, and cortisol; self-reported history of heart disease; participation in a weight-loss program; adherence to a restrictive/therapeutic diet in the past 3 months; self-reported weight changes (greater than 3.0 kg); planned or recent bariatric surgery; consumption of weight altering medications and/or nutritional supplements that provide weight gain/loss in the past 6 months; clinical diagnosis of any mental disorder; current use of mental health medications; for females, pregnant, nursing, or with self-declared intention to become pregnant during the trial; and, current and anticipated enrollment in another research study.

The self-declared items in the exclusion criteria will be obtained using a questionnaire that will be provided during the registration to the trial. A licensed physician will be present during the screening visits to determine if a prospective participant is eligible to join the trial.

### 2.4. Genetic counseling

All participants, regardless of whether they will soon be randomized to either the intervention or control arm, will undergo pre-and post- genetic test counseling sessions. A genetic counselor will meet with the participants for a two-part, interactive, one-on-one virtual counseling. The pre-genetic test counseling will be scheduled a few days after the DNA sample collection and before genotyping of the samples. During this session, the genetic counselor will provide genetic education to the participants, with topics such as basic information about genes, chromosomes, genetic mutations, environmental and genetic interactions, and genetic testing procedures. The genetic counselor will also discuss the impact and potential emotional and psychological concerns that may arise from the result of the genetic test.

The post-genetic test counseling session will be conducted 2 weeks after the pre-test genetic counseling, in time for the release of the genetic testing results of the participants. The genetic counselor will disclose the genotyping results to the participants, along with psychosocial counseling to assist the participants in adapting the new information.

### 2.5. Sample size calculation

To address the primary research question, at least 52 participants (*n* = 26 per group) are needed to detect a clinically meaningful difference of 5% weight loss after 6 months of intervention, assuming 80% power, an alpha of 5%, and a 0.25 SD in the main outcome variable (31). We aim to recruit 62 participants (*n* = 31 per group) to account for the potential dropout rate of 20%.

### 2.6. Intervention

#### 2.6.1. Intervention arm

Participants in the intervention arm will receive the MyGeneMyDiet^®^ Recommendations. It is a gene-based nutrition and lifestyle recommendation developed by the research team by incorporating genetic information based on the result of a genetic test into the standard recommendations for weight management. The MyGeneMyDiet^®^ Recommendations are customized diet and lifestyle advice that will be based on the participant’s anthropometric data (BMI, waist circumference, body fat percentage), biochemical test results, dietary intake, physical activity level, and genetic risk profile.

Decision trees and coded messages were generated by simulating possible genotypes, anthropometric data, results of clinical tests, dietary intake, and physical activity level (Supplementary Table S1; Supplementary Figures S1–S4). A Scientific Advisory Board consisting of nutrition and dietetic professionals, genetic counselors, and a lifestyle medicine physician guided the team in developing the recommendations. The overview of the weight management recommendations among carriers of the risk alleles for *FTO* rs9939609, *UCP1* rs1800592, and *TCF7L2* rs7903146 is described in Table 1.

**Table 1:** Overview of the MyGeneMyDiet^®^ recommendations.

Outcomes	Genetic polymorphisms	Standard recommendations (control arm)	MyGeneMyDiet^®^ recommendations (Invervention arm)
Weight, BMI, and physical activity	*FTO* rs9939609	Achieve and maintain a normal BMI 20–40 min of daily moderate-intensity aerobic physical activity (32)	Achieve and maintain a normal BMI. 30–60 min of daily moderate-intensity aerobic physical (33)
Calorie requirement	*UCP1* rs1800592	Follow the recommended daily caloric intake based on desirable body weight (DBW) and physical activity level (PAL) (34)	Reduce 150 kcal from the recommended daily caloric intake (35)
Dietary fat intake	*TCF7L2* rs7903146	25–30% of the recommended caloric intake from fat (36)	15–20% of the recommended caloric intake from fat (18)

Should the participant in the intervention group have more than one genetic polymorphism, all the corresponding MyGeneMyDiet^®^ recommendations (based on the risk alleles they carry) will be given.

The intervention will be delivered by trained and registered nutritionist-dietitians who will meet with the participants during online counseling sessions in months 0, 3, 6, and 12. These sessions will track the compliance with the meal and exercise plan, and to adjust the caloric and weight loss goals of the participants.

#### 2.6.2. Control arm

Participants in the control arm will be provided with the standard recommendations for weight management. For this study, the standard recommendations are based on the Philippines’ Nutrition Practice Guidelines (NPG) for the Screening and Management of Obesity, and other population-based recommendations including the 2012 Nutritional Guidelines for Filipinos (37), the *Pinggang Pinoy* (Filipino food plate model) dietary recommendations (38), and the WHO 2020 Guidelines on Physical Activity. Except for the customized recommendation based on alleles for *FTO* rs9939609, *UCP1* rs1800592, and *TCF7L2* rs7903146, the advice for the control arm will be based on the participant’s anthropometric data, biochemical test results, dietary intake, and physical activity level.

Participants in the control arm will also receive online nutrition counseling from trained and registered nutritionist-dietitians in months 0 (baseline), 3, 6, and 12. As part of the after-trial care, participants in the control arm will receive the corresponding MyGeneMyDiet^®^ Recommendation for Weight Management after the month 12 visit.

### 2.7. Outcomes

The primary outcomes will be the difference in weight, BMI, waist circumference, and body fat percentage between the arms after both the active and inactive phases. Secondary outcomes will include improvement in dietary intake, eating behavior, physical activity levels, glycated hemoglobin, and blood lipid profile. In addition, stages and motivation for weight loss and the knowledge and perceptions in nutrigenomics and genetic testing will be evaluated.

### 2.8. Randomization and allocation

All participants will be stratified by BMI using the World Health Organization (WHO) cutoff. Overweight (BMI of 25–30 kg/m^2^) and obese (BMI of >30 kg/m^2^) adults will be randomly distributed into blocks of 4, 6, and 8. Within these blocks, each participant will be randomly allocated to the intervention group (MyGeneMyDiet^®^) or control (standard weight management recommendation) using a Random Allocation Software.^1^ Two research staff will perform the randomization.

During randomization, 62 identical (31 for each treatment) sequentially numbered, opaque sealed envelopes (SNOSE) will be prepared. The sequential numbering will be synchronized with the online registration and will be used to designate participant study numbers. The randomization allocation will be concealed from the participants and the rest of the researchers involved in the month 0 visit.

### 2.9. Data collection methods

Study outcomes will be assessed at five time points: baseline (month 0), and months 3, 6, 9, and 12. Table 2 provides an overview of data collection and outcome measures.

**Table 2:** MyGeneMyDiet^®^ study data collection and outcome measures.

Outcome measures	Active phase			Inactive phase	
	Baseline (Month 0)	Month 3	Month 6	Month 9	Month 12
Demographic information	X				
Anthropometry (weight, BMI, waist circumference, hip circumference, percent body fat)	X	X	X	X	X
Dietary intake (3-day food record)*	X	X	X	X	X
Blood chemistry analysis (glycated hemoglobin, lipid profile)	X	X	X	X	X
Physical activity level (IPAQ-SF)	X	X	X	X	X
Eating behavior (TFEQ R-18)	X	X	X	X	X
Motivation for weight loss (SP-Weight Loss)	X	X	X	X	X
Knowledge and perception of nutrigenomics	X		X		X

* Monthly 3-day food records are also collected from the participants, in addition to the food records collected on months 0, 3, 6, 9, and 12.

The research team will conduct the screening and subsequent data collection sessions at the Food and Nutrition Research Institute, Taguig City, Philippines. Pre-session visits will be conducted 2 weeks before the online nutrition counseling sessions.

#### 2.9.1. Anthropometric measurements

Body weight and body fat percentage will be determined using a body composition analyzer (Tanita-MC78U, Tanita Corporation, Japan). Height will be measured using a stadiometer (Seca 217, Seca, Germany) while waist and hip circumference will be obtained using a non-stretchable tape measure (Seca 203, Seca, Germany). Anthropometric measurements will be done twice following a standard procedure. A third measurement will be taken if the difference between the two measurements is greater than 0.3 kg for weight, 0.5 cm for height, and 0.5 cm for waist circumference. The average of all the readings will be taken as the final estimated value of the measurement. BMI will be derived by dividing the weight in kilograms by height in meters squared. The percent body fat (BF%) will be assessed according to the chart by Gallagher et al. (39).

#### 2.9.2. Dietary intake

On a monthly basis, participants will be asked to complete a three-day food diary to estimate their mean energy and macronutrient intake while enrolled in the trial. The food record will consist of two non-consecutive weekdays and one weekend. In total, the participants will accomplish 12 sets of food diaries during the trial.

Proper recording of food and beverage intake and estimating food portion sizes will be explained to the participants during the orientation session. The food diary must reflect all the food and beverages consumed by the participants within the specified periods, the description of each food items (type, variety, brand name), amounts, the time of the day the meal is taken, cooking method (e.g., boiled, fried, broiled), and the source of the food (e.g., home-cooked or takeaway). A food diary form (printed or in electronic form, depending on the participant’s preference) will be distributed during the pre-session visits or approximately 2 weeks before the online follow-up sessions.

Checking and verification to ensure the completeness and correctness of the recorded intake will be done by the nutritionists of the research team, 2 weeks before the online nutrition counseling session. This will provide sufficient time for the nutritionists to analyze the dietary intake of the participants. Energy and macronutrients will be computed using the Philippine Food Composition Tables (FCT). Other foreign FCTs will be used for the items not found in the local FCT.

#### 2.9.3. Biochemical data

Blood samples will be collected during the specified time points (months 0, 3, 6, 9, and 12). The participants will be asked to undergo 10–12 h of fasting before specimen collection. A phlebotomist will collect approximately 5 mL of blood for the analysis of glycated hemoglobin and lipid profile.

#### 2.9.4. Physical activity

The International Physical Activity Questionnaire – Short Form (IPAQ-SF) will be used to measure the level of physical activity of the participants. The short version has seven questions to assess the type and intensity of physical activity that individuals do as part of their daily lives and to estimate the total physical activity in metabolic equivalent (ME).

#### 2.9.5. Eating behavior

The Three-Factor Eating Behavior Questionnaire (TFEQ-R18) (40) will evaluate the eating behavior of the participants. The questionnaire will capture the current dietary practices and aspects of eating behavior such as cognitive restraint eating (conscious restriction of food intake), uncontrolled eating (tendency to eat more than usual due to loss of control over intake accompanied by subjective feeling of hunger), and emotional eating (inability to resist emotional cues).

#### 2.9.6. Level of motivation to change

The Stages (S-Weight) and Process (P-Weight) of Change for Weight Loss Questionnaire will be used to assess the participants’ motivation to lose weight. The S-Weight consists of five mutually exclusive items that will allocate the participants to one of the five stages of change: pre-contemplation, contemplation, preparation, action, and maintenance (41, 42). The P-Weight is a 34-item questionnaire that measures the four processes of change: emotional re-evaluation, weight management actions, environmental restructuring, and weight consequences evaluation (41–44).

#### 2.9.7. Knowledge and perceptions on genetic testing and nutrigenomics

Participants will be asked to complete a survey designed to assess awareness, perceptions, and self-efficacy toward genetic testing and nutrigenomics. Written permission from the main author of the questionnaire (45) was sought before using it for this study.

### 2.10. Statistical analysis plan

Outcome data will be reported following the Consolidated Standard Reporting Trials (CONSORT) guidelines (46). An intention-to-treat analysis will be employed.

The mean and standard deviation (SD) will be used to report continuous variables while percentages will be used for categorical variables. Estimates of the different sources of attrition bias will be conducted using two-way analysis of variance (ANOVA) models. Student *t*-test will be used to compare the differences in means between the intervention and the control group for the primary and secondary outcome measures. One-way ANOVA will be used if the comparison of multiple subclasses is warranted, with Dunn’s multiple comparisons *post-hoc* test. Linear mixed models will be used to determine changes from baseline among the different study outcomes, with participant number retained as random effect. A two-sided alpha of 0.05 will be used for hypothesis testing.

### 2.11. Monitoring

Adverse events and withdrawals will be monitored by the research team. Any adverse events and deviations will be reported to the FNRI Institutional Ethics Review Committee and included in the continuing ethics review of the trial.

## 3. Discussion

This trial aims to provide evidence to support a strategy of gene-based nutrition lifestyle intervention to manage weight loss. It will incorporate disclosure of genetic risk and recommendations based on the genetic profile of the participants.

To the best of our knowledge, this will be the first gene-based nutrition and lifestyle intervention that will adopt an intensive gene-based nutrition counseling, followed by an “inactive” period of 6 months where a “free-living” state is simulated. Along with the goals of evaluating the effectiveness of gene-based nutrition and lifestyle advice, this trial will also raise the awareness of the Filipino population regarding nutritional genomics. It acknowledges the vital role of genetic counselors in guiding the population in making informed choices upon learning their genetic risks, particularly so that all participants are carriers of at least one (or a combination) of the risk alleles for *FTO* rs9939609, *UCP1* rs1800592, and *TCF7L2* rs7903146. This trial will play an important role in leveraging a unique multidisciplinary approach to weight management that involves nutrition professionals and genetic experts.

Limitations of the study include (1) limited applicability of the findings to other populations since the trial will be conducted among overweight/obese Filipino adults only; (2) the recommendations are based only on three genetic polymorphisms, and it is likely that there are other genetic loci that may pose risks to unhealthy weight gain that will not be tackled in this study, and; (3) the intervention will only entail counseling/education and no standardized meals will be given.

## 4. Ethics and dissemination

### 4.1. Research ethics approval

This protocol was approved by the Food and Nutrition Research Institute (FNRI) Institutional Ethics Review Committee (FIERC-2021-001). In case protocol amendments are required, approval from the FIERC will be obtained before applying any changes. The current version of the protocol is version 7 (dated 2022-04-18).

### 4.2. Confidentiality

Substitute codes will be used to protect the identity of participants. A designated secured cabinet and password-protected central database will be used to store the trial documents. All recorded video conversations on Zoom will be downloaded immediately after each session and will be deleted from the Cloud once the recording is assured to have been saved in a password-protected external drive. Only the research team members who performed the randomization and allocation have access to the data collection and storage devices.

### 4.3. Dissemination policy

The research findings will be submitted to and published in peer-reviewed journals.

## Data availability statement

The original contributions presented in the study are included in the article/Supplementary material, further inquiries can be directed to the corresponding author.

## Ethics statement

The studies involving human participants were reviewed and approved by the FNRI Institutional Ethics Review Committee (FIERC-2021-001). The participants provided their written informed consent to participate in this study.

## Author contributions

JN wrote the manuscript and the primary investigator of this study. JN, JL, and DR designed the trial. JN and MR provide overall administrative support to the study. JN, MR, GG, and DR spearhead the clinical and genomic aspects of the trial. RF, MM, NS, and JL spearhead the nutrition and dietary components of the study. AD, RF, MM, NS, JC, and MF contributed to the development of the trial protocol. All authors will be involved in the collection of data. All authors contributed to the critical review of this manuscript for intellectual content and approved the submitted version. All authors substantially revised, read, and approved the final manuscript.

## Funding

The MyGeneMyDiet^®^ trial is part of the Interventions using Genomics-based Strategies (InGeSt) Research Program of the Department of Science and Technology – Food and Nutrition Research Institute funded through the Locally Funded Project (LFP) of the Philippines’ Department of Budget and Management (DBM).

## Conflict of interest

The authors declare that the research was conducted in the absence of any commercial or financial relationships that could be construed as a potential conflict of interest.

## Publisher’s note

All claims expressed in this article are solely those of the authors and do not necessarily represent those of their affiliated organizations, or those of the publisher, the editors and the reviewers. Any product that may be evaluated in this article, or claim that may be made by its manufacturer, is not guaranteed or endorsed by the publisher.

## Footnotes

^1^ https://www.random-allocation-software.software.informer.com/2.0/
