# Supplementary material for: A study protocol for a pilot randomized controlled trial to evaluate the effectiveness of a gene-based nutrition and lifestyle recommendation for weight management among adults: the MyGeneMyDiet® study
Source: Front Nutr. 2023 Aug 22;10:1238234. doi: 10.3389/fnut.2023.1238234 (PMC10477364; doi:10.3389/fnut.2023.1238234)
Supplement: Supplementary file 1 [file Data_Sheet_1.pdf]

Supplementary Material

A study protocol for a randomized controlled trial to evaluate the effectiveness of a gene-based nutrition and lifestyle recommendation for weight management among adults: the MyGeneMyDiet® study

Jacus S. Nacis[1,2*], Jason Paolo H. Labrador[1], Diana Glades D. Ronquillo[1], Marietta P. Rodriguez[1], Aurora Maria Francesca D. Dablo[1], Ruby D. Frane[1], Marilou L. Madrid[1], Noelle Lyn C. Santos[1], Julianne Janine V. Carrillo[1], Mikko Glen Fernandez[1], Gerard Bryan L. Gonzales[2,3]

[1]Department of Science and Technology – Food and Nutrition Research Institute (DOST-FNRI), General Santos Avenue, Bicutan, Taguig City 1631 Metro Manila, Philippines

[2]Division of Human Nutrition and Health, Wageningen University & Research, Wageningen, The Netherlands

[3]Department of Public Health and Primary Care, Faculty of Medicine and Health Science, Ghent University, Corneel Heymanslaan 10 K3, ingang 42, 9000 Gent, Belgium

*** Correspondence:**
Corresponding Author
jacusnacis@gmail.com / jacus1.nacis@wur.nl

1 **Supplementary Table 1:** MyGeneMyDiet® recommendations covered concepts on weight loss.

Codes	Covered concepts on weight loss
**Disclosure of Genotype and Risk Information**	
FTO.C.A.1	Disclosure of *FTO* risk genotype response on Obesity
FTO.C.A.2	Disclosure of *FTO* risk genotype response on Physical Activity
FTO.NC.A.1	Disclosure of *FTO* non-risk genotype response on body weight, BMI, waist circumference and percent body fat.
FTO.NC.A.2	Disclosure of *FTO* non-risk genotype response on Physical Activity
UCP.C.A.1	Disclosure of *UCP1* risk genotype response on Energy Balance

UCP.C.A.2	Information on Basal Metabolic Rate (BMR)
UCP.NC.A	Disclosure of *UCP1* non-risk genotype response on Energy Balance
TCF.C.A.	Disclosure of *TCF7L2* risk genotype response to a low-fat diet
TCF.NC.A	Disclosure of *TCF7L2* non-risk genotype response to a low-fat diet
**Goals and Targets for Weight Management**	
FTO.C.B.1	Body Weight and BMI targets for *FTO* risk genotype
FTO.C.B.2	Physical activity level targets for *FTO* risk genotype
FTO.C.B.2.1	Additional Physical activity level targets for *FTO* risk genotype
UCP.C.B	Energy Requirement for *UCP1* risk genotype
TCF.C.B	Fat distribution recommendation for *TCF7L2* risk genotype
S.B.1.1	Healthy Weight Loss Percentages
S.B.1.2	Normal Body Mass Index (BMI) Range and Desirable Body Weight (DBW)
S.B.1.3	Waist Circumference Cut-off points
S.B.1.4	Healthy range for %Body Fat for adult males and females
S.B.2.1	Information on Healthy Weight Loss Equation (Less Energy IN, More Energy OUT)
S.B.2.2	Daily Calorie Deficit for weight loss
S.B.3	Recommended Macronutrient Distribution for weight loss
S.B.4.1	Physical Activity Recommendations for adults
S.B.4.2	Recommendations for Muscle Strengthening Activities for adults

S.B.4.3	Examples of Physical activities and exercises
S.B.4.4	Additional Physical Activity Recommendations for adults
**Portion Control for weight loss**	
S.C.1	Portion Control and Pinggang Pinoy®
**Distribution of Meals**	
S.C.2	Distribution of meals throughout the day and avoidance of skipping meals
**Carbohydrate Intake**	
S.C.3.1	Acceptable Macronutrient Distribution Range for Carbohydrates
S.C.3.2	Enriched and Whole-Grain Products
S.C.3.3	Complex and Starchy Carbohydrates
**Dietary Fiber Intake**	
S.C.4.1	Recommended Dietary Fiber Intake with household measures
S.C.4.2	Daily Vegetable and Fruit Servings
S.C.4.3	Whole Fruits for fiber benefits
**Added Sugars**	
S.C.5.1	Added sugars
S.C.5.2	Sugar-sweetened Beverages
S.C.BC.1	Recommendations for elevated fasting blood glucose
**Protein Intake**	
S.C.6.1	Acceptable Macronutrient Distribution Range for Protein

S.C.6.2	Lean Meat
S.C.6.3	Eggs
S.C.6.4.1	Milk and Dairy Products
S.C.6.4.2	Low-Fat and Fat-Free Food Products
S.C.6.4.3	Information on Lactose Intolerance
S.C.6.5	Protein Alternatives
**Fat Intake**	
S.C.7.1	Acceptable Macronutrient Distribution Range for Fat
S.C.7.2	Foods low in Fat and Cholesterol
S.C.BC.2	Recommendations for elevated blood lipid profile
S.C.7.3	Types of Fats (Saturated, Unsaturated, Trans Fat)
S.C.7.4	Information on Coconut Oil
S.C.7.5	Oils
**Nutrient-dense Foods**	
S.C.8	Nutrient-dense Foods
**Sodium Intake**	
S.C.9.1	Use of salt in food preparation
S.C.9.2	Sodium in natural foods
S.C.9.3	Sodium in processed food items

S.C.9.4.1	Use of condiments
S.C.9.5	Sodium content in food labels
**Gut Health**	
S.C.10.1	Information on gut microbiota and obesity
S.C.10.2	Examples of probiotic food items
**Hydration**	
S.C.11	Hydration and fluid intake
**Physical Activity Recommendations**	
S.D.1.1	Importance of seeking guidance from a Medical Doctor before engaging in a Physical Activity Program
S.D.1.2	Gradual Increase of Physical Activity for start-up
S.D.1.3	Be Active in Your Workplace
S.D.1.4	Be Active at Home
S.D.1.5	Avoidance of Sedentary Activities
S.D.1.6	Safety and Body Signals during exercises
S.D.1.7	Hydration and fluid intake before, during, and after exercises
**Meal replacements for weight loss**	
S.D.2	Meal replacements for weight loss
**Alternative Strategies for weight loss**	
S.D.3.1	Weight loss strategies to avoid (fad diets, use of herbal medicines, or health supplements)

S.D.3.2	Consulting a Registered Nutritionist-Dietitian (RND) for weight management
**Choosing Healthier Foods**	
S.D.4.1	Reading Nutrition Labels
S.D.4.2	Healthy Shopping
**Healthy Cooking**	
S.D.5	Healthier Cooking Methods
**Healthy Lifestyle**	
S.D.6.1	Lifestyle Modification for weight loss
S.D.6.2	Alcoholic Beverages
S.D.6.3	Smoking, Stress, and Sleep
**Mindful eating**	
S.D.7.1	Eat Slowly
S.D.7.2	Use of smaller plates and servings
S.D.7.3	Regular Eating Pattern
S.D.7.4	Prepare Before You Go
S.D.7.5	Eating out
**Towards Behavior Change**	
S.D.8.1	Weight loss as a long-term commitment
S.D.8.2	Set S.M.A.R.T Goals
S.D.8.3	Think of Small Wins

S.D.8.4	Importance of Self-Monitoring
S.D.8.5	Social and Environmental Triggers

2 Supplementary Figures

2.1 Supplementary Figures

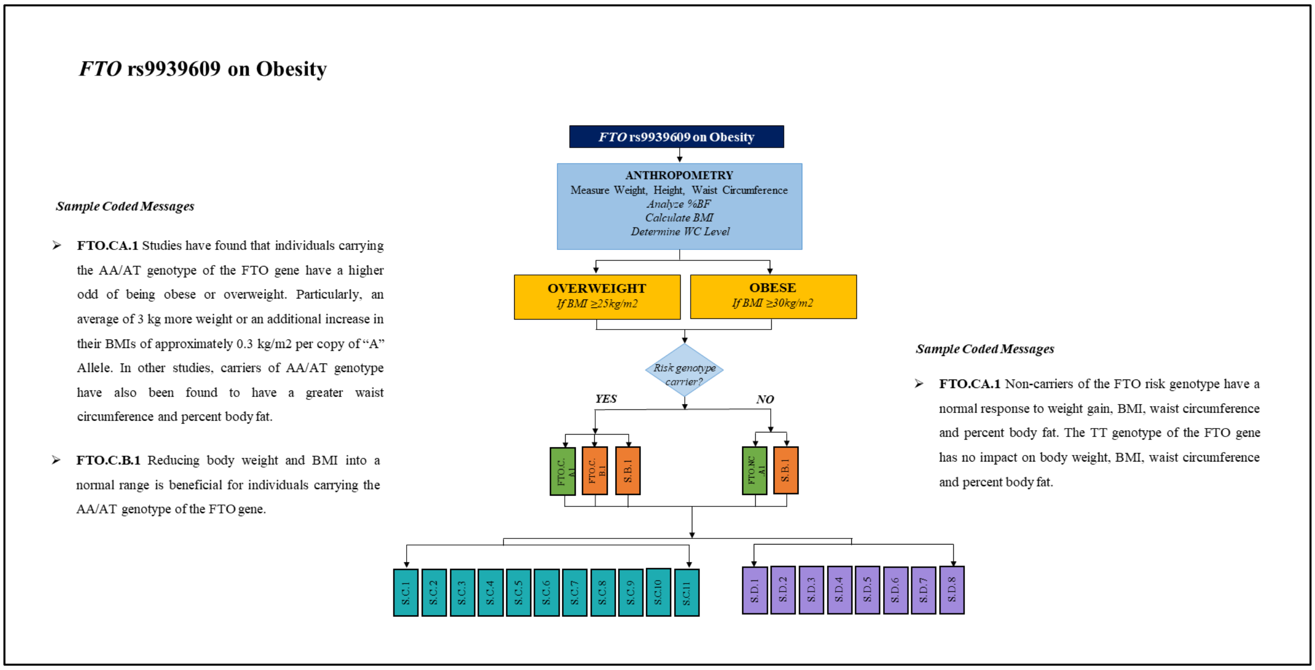

Supplementary Figure 1. Decision trees for the MyGeneMyDiet® Recommendations of *FTO* rs9939606 on obesity with sample coded messages

FTO rs9939609 on Physical Activity

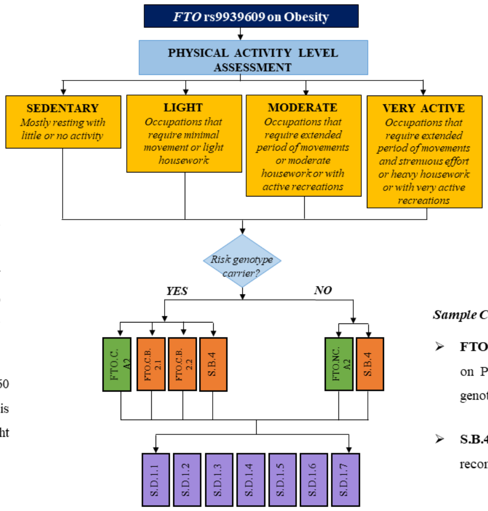

Sample Coded Messages

- **FTO.C.A.2** The role of physical activity and exercise in reducing the susceptibility to obesity is beneficial for FTO AA/AT genotype carriers. Previous studies have shown that enhancing the physical activity level of individuals carrying the AA/AT genotype of the FTO gene can weaken its impact on weight and body composition.
- **FTO.C.B.2.1** An additional 5-10 minutes per day or 30-60 minutes per week on top of the standard recommendation is beneficial to diminish the impact of FTO on body weight and body composition

Sample Coded Messages

- **FTO.NC.A.2** Non-carriers of the FTO risk genotype have normal response on Physical Activity's impact to weight and body composition. The TT genotype of the FTO gene has no impact to your physical activity level.
- **S.B.4.** On average, 20-40 minutes of daily physical activity is recommended for weight loss.

Supplementary Figure 2. Decision trees for the MyGeneMyDiet® Recommendations of *FTO* rs9939606 on physical activity with sample coded messages

UCP1 rs1800592 on Caloric Requirement

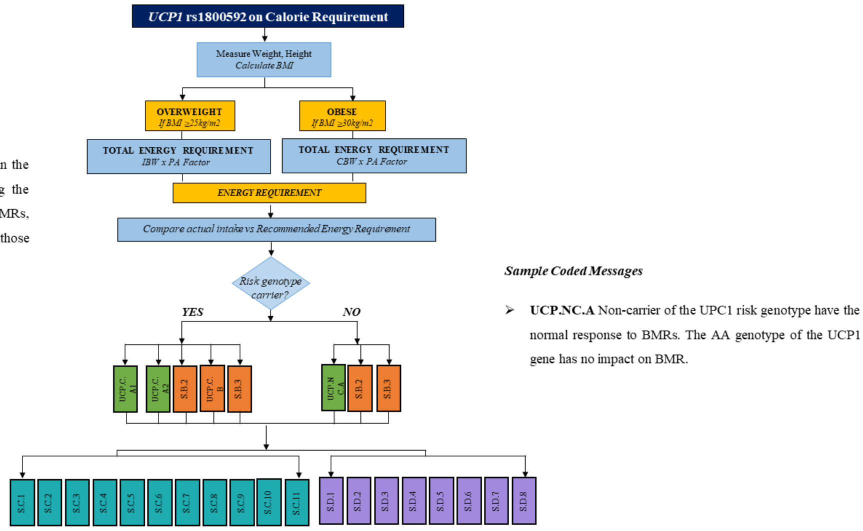

Sample Coded Messages

- **UCP.C.A.1** The UCP1 gene can impact variations in the Basal Metabolic Rate (BMR). Individuals carrying the GG/GA genotype tend to have lower BMRs, approximately 150 calories (about 10%) lower than those with the AA genotype.
- **UCP.C.B.** An additional 150 calories will be deducted to the calculated energy needs to achieve successful weight loss for individuals carrying the GG/GA genotype of the UCP1 gene.

Sample Coded Messages

- **UCP.NC.A** Non-carrier of the UPC1 risk genotype have the normal response to BMRs. The AA genotype of the UCP1 gene has no impact on BMR.

Supplementary Figure 3. Decision trees for the MyGeneMyDiet® Recommendations of *UCP1* rs1800592 on caloric requirement with sample coded messages

TCF7L2 rs7903146 on Dietary Fat Intake

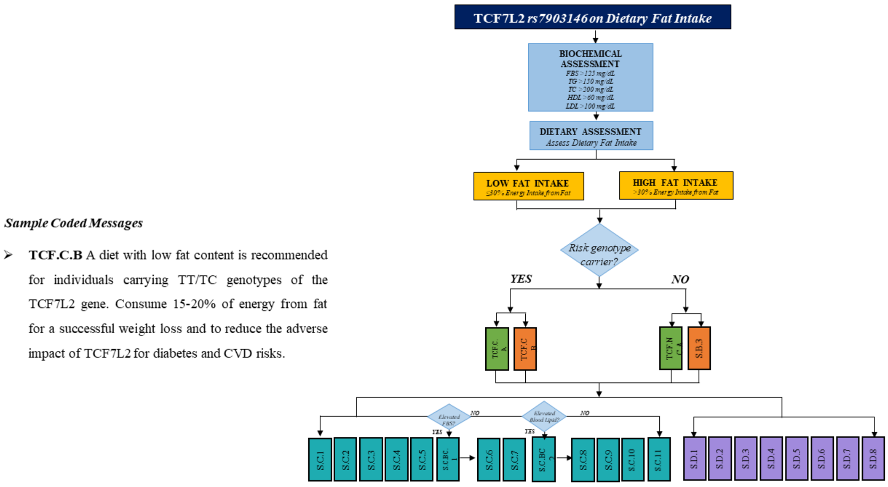

Sample Coded Messages

- **TCF.C.B** A diet with low fat content is recommended for individuals carrying TT/TC genotypes of the TCF7L2 gene. Consume 15-20% of energy from fat for a successful weight loss and to reduce the adverse impact of TCF7L2 for diabetes and CVD risks.

Sample Coded Messages

- **TCF.NC.A** Non-carriers of the TCF7L2 risk genotype have the normal response on dietary fat for weight loss. The CC genotype of the TCF7L2 gene has no impact on dietary fat intake.
- **S.C.7.1** Daily intake of fat should be 25-30% of the total energy requirement.

Supplementary Figure 4. Decision trees for the MyGeneMyDiet® Recommendations of *TCF7L2* rs7903146 on dietary fat intake with sample coded messages
